# Codon Composition in Human Oocytes Reveals Age-Associated Defects in mRNA Decay

**DOI:** 10.3390/ijms26199395

**Published:** 2025-09-26

**Authors:** Pavla Brachova, Lane K. Christenson, Nehemiah S. Alvarez

**Affiliations:** 1 Department of Biomedical and Translational Sciences, Division of Woman and Child Health, Eastern Virginia Medical School, Macon and Joan Brock Virginia Health Sciences, Old Dominion University, Norfolk, VA 23518, USA; 2 Department of Cell Biology and Physiology, University of Kansas Medical Center, Kansas City, KS 66160, USA; 3 Advanced Sequencing Program, Eastern Virginia Medical School, Macon and Joan Brock Virginia Health Sciences, Old Dominion University, Norfolk, VA 23518, USA

**Keywords:** reproductive aging, maternal mRNA decay, codon optimality, oocyte, GV-to-MII, translation

## Abstract

Oocytes from women of advanced reproductive age exhibit diminished developmental potential, but the underlying mechanisms remain incompletely defined. Oocyte maturation depends on translational control of maternal mRNA synthesized during growth. We performed a computational analysis on human oocytes from women <30 versus ≥40 years and observed that mRNA GC content correlates negatively with half-life in oocytes from young (<30 yr) but positively with oocytes from aged (>40 yr) women. In young oocytes, longer mRNA half-life is associated with lower protein abundance, whereas in aged oocytes GC content correlates positively with protein abundance. During the GV-to-MII transition, codon composition stratifies stability: codons that support rapid translation (optimal) stabilize mRNA, while slow-translating codons (non-optimal) promote decay. With reproductive aging, GC-containing codons become more optimal and align with increased protein abundance. These findings indicate that reproductive aging remodels codon-optimality-linked, translation-coupled mRNA decay, stabilizing a subset of GC-rich maternal mRNA that may be prone to excess translation during maturation. Our analysis is explicitly within human reproductive aging; it does not revisit cross-species stability rules. Instead, it shows that sequence–stability relations are reprogrammed with age within human oocytes, including an inversion of the GC–stability association during GV-to-MII transition. Disruption of the normal mRNA clearance program in aged oocytes may compromise oocyte competence and alter maternal mRNA dosage, with downstream consequences for early embryonic development.

## 1. Introduction

The ovary is the first organ in women to exhibit functional decline with age, a process termed reproductive aging, marked by progressive loss in both oocyte quantity and competence, coinciding with a concomitant fall in fertility [1]. Despite the clinical impact of reproductive aging, the cellular and molecular drivers remain incompletely defined. A defining feature is the rising burden of meiotic aneuploidy in oocytes [2,3,4]. Consistent with this, mouse studies indicate that aging disrupts translation of key meiotic regulators, a defect hypothesized to precipitate chromosome-segregation errors [5,6]. Additionally, degradome profiling shows that meiosis-coupled maternal mRNA decay is impaired in aged human (and mouse) oocytes [7]. Because translation in oocytes is intimately linked to the stability and timely clearance of maternal mRNA (CCR4–NOT/BTG4-dependent deadenylation that prevents premature translational activation and promotes meiosis-coupled mRNA decay) defects in translational control are expected to disrupt RNA clearance and oocyte maturation in both mouse and human oocytes [8,9].

Oocyte growth is marked by intense transcription; the resulting maternal mRNA are either translated to build the oocyte proteome or sequestered in repressed ribonucleoprotein stores for later use [10,11,12]. In fully grown mouse oocytes, transcription shuts down and does not restart until zygotic genome activation; consequently, maturation and early development rely on stored maternal mRNAs [12]. As stored mRNA are recruited to ribosomes, they undergo poly(A)-tail shortening, destabilization, and degradation through translation-coupled mRNA decay, thereby linking the act of translation directly to mRNA stability; genetic evidence further shows that the RNA acetyltransferase NAT10 promotes this program by sustaining CCR4-NOT activity through ac4C on Btg4/Cnot6l/Cnot7 transcripts in mouse oocytes [13,14,15,16]. Thus, features that govern translation (e.g., codon composition and GC content) also set mRNA half-life; precise coordination of translation and decay is a key determinant of oocyte quality and a likely point of vulnerability during reproductive aging.

The sequence of mRNA encodes regulatory information that shapes translation and RNA stability [15,17,18,19]. The 3′ UTR, including the choice of poly(A) extension sites, governs the recruitment of RNA-binding proteins and microRNA, while the poly(A)-tail length also modulates both stability and translational output [18,20,21,22]. Oocyte-specific RNA regulators such as LSM14B (storage granules/MARDO) and ZAR1/2 (polyadenylation control) also gate when maternal mRNA remain stored versus activated during oocyte maturation [23]. Consistent with selective recruitment, eIF4E1B preferentially activates maternal mRNA bearing GC-rich 5′-UTR motifs during the oocyte to embryo transition [24]. Beyond these regions, the coding sequence also enforces translation-dependent control via codon composition, codon optimality, where enrichment for “optimal” codons stabilizes mRNA, increases abundance, enhances translation efficiency, and associates with longer poly(A) tails, whereas “non-optimal” codons do the opposite [25,26,27,28,29,30,31,32,33,34]. Mechanistically, non-optimal codons can impede translation initiation [35], slow elongation, and shorten mRNA half-life [25,36]. These codon-dependent stability rules, collectively termed codon-optimality–mediated mRNA decay (COMD), are observed across model systems and in human cell lines [25,26,37,38,39]. Thus, although the coding sequence specifies protein identity, it also tunes protein levels by dictating mRNA stability through COMD [34]. Interestingly, inosine mRNA modifications, which are perceived as guanine residues by the translational and mRNA decay machinery, are highly enriched in oocytes, particularly at the codon wobble position [40]. Inosine mRNA modifications in codons provide a mechanism to functionally increase the GC-content of transcripts, potentially marking them for clearance. This is particularly relevant as the core maternal mRNA decay machinery, driven by factors like the CNOT6L deadenylase, is mechanistically coupled to the inosine modification landscape. Indeed, in oocytes lacking CNOT6L, in which general mRNA decay is impaired, inosine-modified transcripts are nevertheless cleared, suggesting that inosine serves as a mark for a parallel, CNOT6L-independent mRNA decay pathway [14,41].

Whether codon composition regulates translational efficiency and mRNA stability in human oocytes, and how this regulation changes with reproductive aging, remains largely unexplored. Our study profiles coding-sequence features across the GV-to-MII transition in oocytes from reproductively young (<30 years) and aged (≥40 years) women to test whether codon usage influences mRNA stability and whether translation-coupled mRNA decay is altered with age. Results indicate that reproductive aging amplifies the maternal mRNA-decay program, sharpening both stabilization and destabilization of selected transcripts. In young oocytes, GC-rich codons exhibit lower codon optimality and shorter mRNA half-lives yet associate with higher protein output, consistent with active translation-coupled decay. With age, GC-rich codons gain optimality and GC-rich transcripts acquire longer half-lives, indicating a shift in codon-optimality-linked stability. These age-dependent changes in codon optimality are poised to recalibrate translational output and oocyte developmental competence. Because the maternal mRNA repertoire preloaded into the zygote shapes zygotic genome activation, such shifts may alter the timing and robustness of ZGA, and newly identified MARTRE proteins in zygotes protect long poly(A) tails by dampening CCR4-NOT, thereby sustaining translation of maternal mRNA [42,43,44].

A recent cross-species study in five mammals reported that greater GC content associates negatively with maternal mRNA stability, whereas codon usage and 3′-UTR length associate positively [19]. Building on that baseline, we test whether these sequence-encoded stability rules are preserved or reprogrammed with reproductive aging within human oocytes. We find an age-dependent inversion of the GC–stability relationship and an age-restricted redefinition of optimal codons during the GV-to-MII transition, with codon stability coefficients aligning positively with protein output only among age-altered transcripts. These observations indicate that reproductive aging, rather than species differences, remodels codon-optimality-linked, translation-coupled RNA decay in human oocytes.

## 2. Results

### 2.1. Age-Associated Changes in mRNA Half-Life

**Figure 1:**
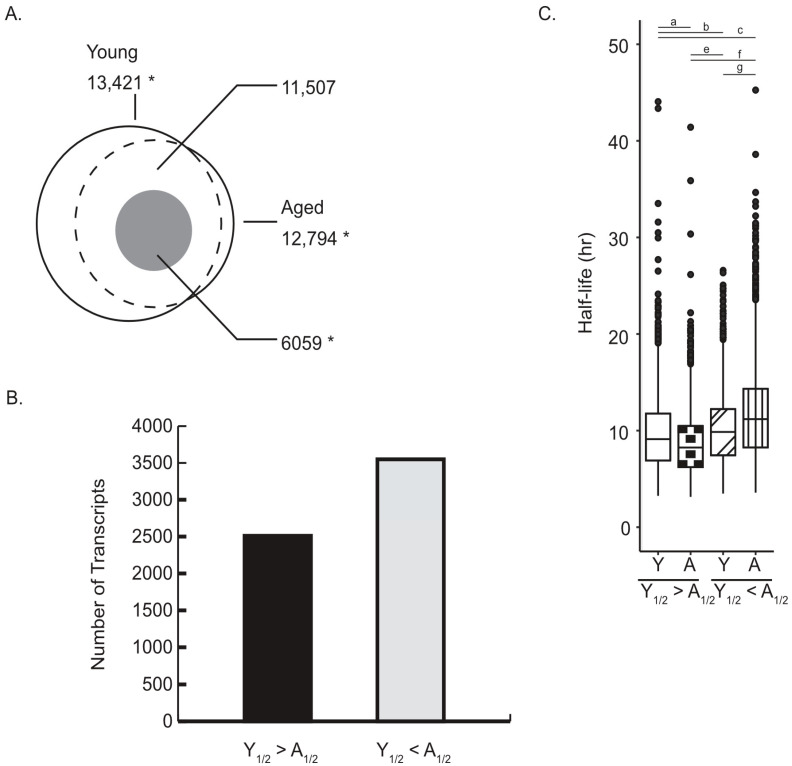
Reproductive aging alters mRNA half-life during oocyte in vitro maturation. (**A**) Venn diagram showing the number of protein-coding transcripts in reproductively young (<30 years; 13,421) and aged (≥40 years; 12,794) oocytes that fit an exponential decay model (*p* < 0.05, *t*-test). The dotted overlap represents the 11,507 shared transcripts present in both groups, and the gray overlay highlights the 6059 shared transcripts with significantly different half-lives between age groups (* *p* < 0.05, *z*-test). Transcripts with shorter half-life in aged oocytes satisfy Y_1/2_ > A_1/2_, whereas those with longer half-life in aged satisfy Y½ < A½ (see panel (**B**)). (**B**) Bar chart showing direction of half-life changes for the 6059 differentially stable transcripts. The 2510 transcripts with a half-life (_1/2_) that is greater in young (Y) versus aged (A; i.e., Y_1/2_ < A_1/2_) are depicted by the black bar): these transcripts have shorter half-lives in aged oocytes. The 3549 transcripts that have longer half-lives in aged oocytes Y_1/2_ < A_1/2_ are depicted by the gray bar ( KS test *p* < 0.05 for indicated comparisons; a, b, c, e, f, g). (**C**) Boxplots comparing half-life distributions for each group in young (Y) and aged (A) oocytes. For transcripts with Y_1/2_ < A_1/2_, median half-life decreased from 9.1 h (Y) to 8.2 h (A). For transcripts with Y_1/2_ < A_1/2_, median half-life increased from 9.9 h (Y) to 11.2 h (A).

During the oocyte GV-to-MII transition, changes in mRNA abundance are due to altered mRNA stability rather than mRNA synthesis. To determine the influence of reproductive aging on mRNA half-life, we analyzed publicly available human oocyte RNA-seq data from reproductively young and aged women (young: <30 years; aged: ≥40 years) [45]. Oocyte transcripts accumulate during the growth phase and are stored in a dormant, untranslated form until meiotic maturation, when they become recruited for translation and degradation in a process called translational mRNA decay [11,13,14]. We therefore focused our decay analysis on protein-coding transcripts that have the potential to be regulated by translational mRNA decay. Previous work has established that a significant number of mammalian transcripts follow an exponential decay pattern [46]. Using an mRNA decay normalization strategy, we fit the oocyte poly(A) RNA sequencing data to an exponential decay curve to calculate mRNA half-life (see Methods). In young oocytes, 13,421 transcripts fit the decay model, while aged oocytes yielded 12,794 transcripts. Of these, 11,507 were shared between the two age groups, and a subset (6059 transcripts, gray overlay, Figure 1A) exhibited significant differences in half-life (_1/2_) with aging (Figure 1A). Of the decay-fit sets, 1914 transcripts were unique to young oocytes and 1287 were unique to aged oocytes; 11,507 transcripts were shared. For these 6059 transcripts, 2510 transcripts are more stable in oocytes from young women (Y_1/2_ > A_1/2_) and 3549 transcripts became more stable with age (Y_1/2_ < A_1/2_; Figure 1B). In young oocytes, transcripts with shorter half-lives in aged oocytes had a median half-life of 9.1 h, while those with a longer half-life in aging had a median of 9.9 h. In aged oocytes, the same groups showed a more pronounced separation, with median half-lives of 8.2 and 11.2 h, respectively (Figure 1C). These observations are consistent with human degradome analyses showing attenuated meiosis-coupled mRNA clearance with advancing maternal age [7].

### 2.2. GC Content of mRNA Positively Correlates with Half-Life During Reproductive Aging

**Figure 2:**
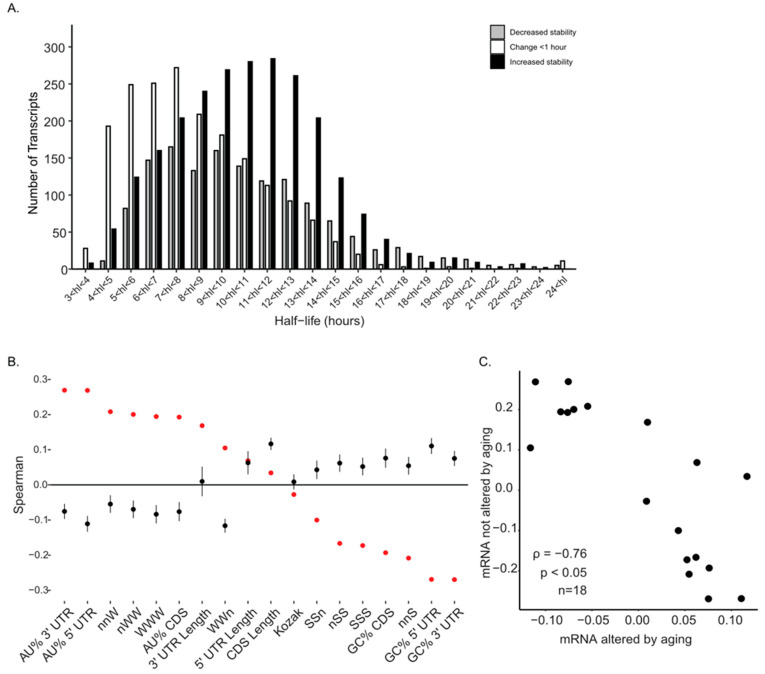
GC content of mRNA positively correlates with half-life during reproductive aging. (**A**) Histogram representing half-life values of decreasing, no change and increasing stability at 1-hour intervals during reproductive aging. (**B**) Mean spearman correlations between mRNA half-life and mRNA features derived from the 1-hour bins in panel (**A**) for mRNA not altered by aging (red dots, mean ± SEM; error bars reside in the dot) and for mRNA altered by aging (black dots, mean ± SEM). nSS, nnS, SSS, SSn are IUPAC codons for GC (SS)-containing codons. nWW, nnW, WWW, WWn are IUPAC codons for AU (WW)-containing codons (Spearman ρ; mean ± SEM). (**C**) Spearman correlation between the feature values of B; ρ, Spearman correlation; *p*, *p*-value; *n*, number of features.

To examine how mRNA sequence composition relates to stability changes with age, transcripts were grouped into three categories: those with less than a one-hour difference in half-life between young and aged oocytes, those with decreased half-life in aged oocytes, and those with increased half-life in aged oocytes (Figure 2A). Most transcripts not altered by aging had half-lives under eight hours. Among transcripts with half-lives greater than eight hours in aged oocytes, the majority were stabilized during aging. In human IVM, GVBD is typically observed approximately 6–8 h after maturation begins, and oocytes remain in MI for roughly ~14 h before reaching MII [47,48]. Pathway analysis revealed that mRNA with increased half-life during aging were enriched in RNA processing, mitotic processes, microtubule organization from the centrosome, and DNA repair pathways (Supplemental Figure S1). Transcripts with decreased half-life during aging were enriched in pathways related to mitochondrial processes, mitochondrial translation, neddylation and SUMOylation, tRNA processing, and DNA repair (Supplemental Figure S2). These patterns are age-contingent within human oocytes and further research would be needed to derive cross-species general rules.

We next performed a correlation analysis between mRNA sequence features and mRNA half-life to identify the drivers of these stability patterns. For transcripts whose stability was unaffected by aging, we observed a strong and positive correlation between AU-rich features (including AU content in the UTRs and AU-containing codons) and mRNA half-life. Conversely, GC-rich features were negatively correlated with half-life in this group (Figure 2B, red dots). In striking contrast, this relationship was inverted for the population of transcripts whose mRNA half-life was altered by aging. In this group, GC content became positively correlated with stability, while AU content showed a negative correlation with half-life (Figure 2B, black dots). This fundamental shift in stability rules was statistically significant, as the correlation patterns between the unchanged and altered transcript groups were strongly and negatively related (ρ = −0.76, *p* < 0.05, Spearman; Figure 2C).

### 2.3. GC Content Reveals a Difference Between mRNA Half-Life and Protein Abundance

**Figure 3:**
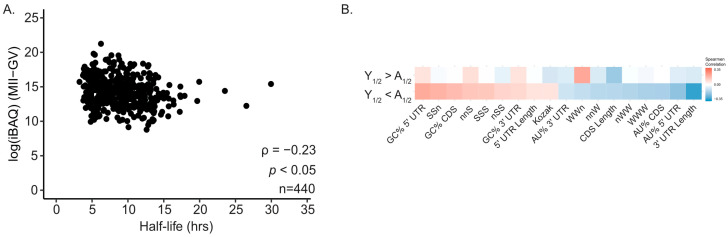
GC/AU content reveals differential correlations between mRNA half-life and protein abundance. (**A**) Scatter plot illustrates relationship between protein abundance (log(iBAQMII—iBAQGV)) and mRNA half-life (reproductively young) from genes (*n* = 440) with a single protein coding isoform (analyses restricted to single protein-group genes, *n* = 440); ρ, Spearman correlation; *p*, *p*-value. (**B**) Spearman correlation between protein abundance (iBAQ, MII-GV) and mRNA features from corresponding mRNA.

To assess how mRNA stability relates to protein production, we compared half-life values with protein abundance measurements obtained from mass spectrometry data during the GV-to-MII transition in human oocytes [49]. We restricted RNA–protein comparisons to genes yielding a single protein group in the proteomics dataset (*n* = 440) to avoid isoform ambiguity. Across all analyzed transcripts, there was an inverse relationship between mRNA half-life and protein abundance (ρ = −0.23, *p* < 0.05; Figure 3A), indicating that transcripts with higher protein output generally had shorter half-lives. In line with this observation, single-cell proteomics of human oocytes with advanced maternal age reports decreased abundance of proteostasis and meiosis proteins despite complex transcript changes [50].

Next, we explored whether the sequence features of the age-affected transcript groups were associated with this translational output. We performed a correlation analysis between the mRNA features and protein abundance for the transcripts that either gained or lost stability with age (Figure 3B). For the group of transcripts that became more stable in aged oocytes (Y_1/2_ < A_1/2_), we found a strong and positive association between GC-rich sequence features and protein abundance. Conversely, AU-rich features in this same group were negatively correlated with protein abundance. In contrast, for transcripts that became less stable during aging (Y_1/2_ < A_1/2_), these correlations were substantially weaker.

### 2.4. Age-Associated Changes in Codon Optimality

**Figure 4:**
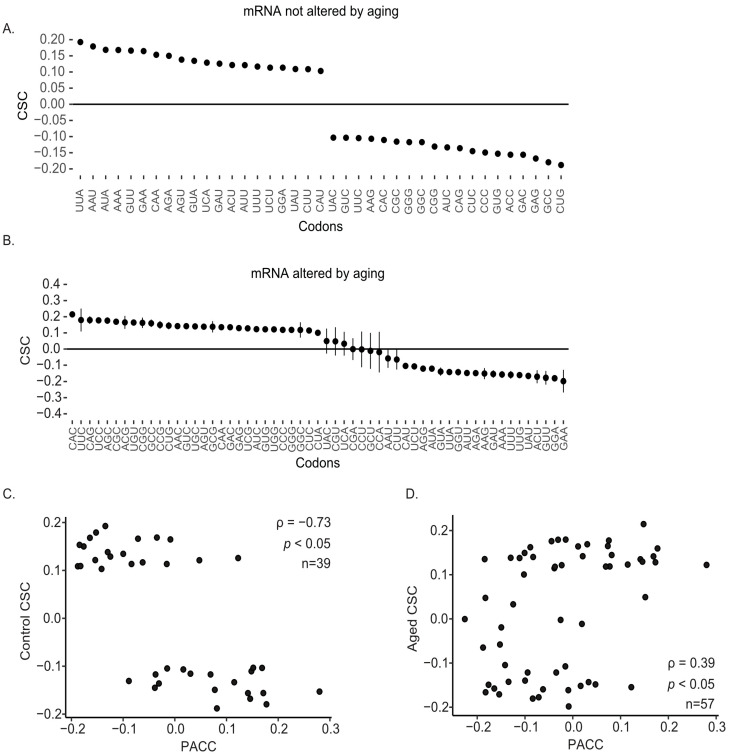
Reproductive aging in human oocytes alters codon optimality. Codon stability coefficients (CSC) are an average of CSC values from each 1-h bin from Figure 2A. CSC values between −0.1 and 0.1 were filtered out prior to averaging; error bars represent SEM. (**A**) CSC from mRNA not altered by reproductive aging. (**B**) CSC from mRNA altered by reproductive aging. Scatter plot comparing the CSC from unaffected (**C**) mRNA (panel (**A**)) and affected (**D**) mRNA (panel (**B**)) to protein abundance codon correlation (PACC); ρ, Spearman correlation; *p*, *p*-value; *n*, number of codons in common.

We examined codon optimality by calculating codon stability coefficients (CSC) for transcripts either unaffected or affected by reproductive aging [25]. For mRNA unaffected by aging, optimal codons were predominantly AU rich, with 19 of 20 optimal codons enriched for AU content (Figure 4A). In contrast, for mRNA whose stability was altered by aging, optimal codons were mainly GC rich, with 24 of 29 optimal codons containing GC nucleotides (Figure 4B). CSC values for aging-affected transcripts were negatively correlated with those from unaffected transcripts (ρ = −0.62, *p* < 0.05; Figure 4C).

To explore how codon usage relates to translation, we calculated the correlation between codon stability coefficients (CSC) and protein abundance differences (MII—GV iBAQ values), hereafter abbreviated as the protein abundance codon correlation (PACC). For unaffected transcripts, CSC and PACC values were negatively correlated (ρ = −0.73, *p* < 0.05), while for aging-affected transcripts, the correlation was positive (ρ = 0.39, *p* < 0.05; Figure 4D).

## 3. Discussion

Within the context of reproductive aging, our data indicate that reproductive aging amplifies existing decay programs while redistributing which transcripts are stabilized or cleared, rather than introducing new programs. Of the 11,507 transcripts shared between age groups, 6059 showed age-dependent half-life differences, 3549 increased and 2510 decreased, demonstrating that reproductive aging redistributes which messages are stabilized or cleared (Figure 1A–C). The inversion of GC–stability correlations among age-altered transcripts (ρ between feature sets = −0.76, *p* < 0.05; Figure 2B,C), the AU to GC shift in optimal codons (Figure 4A,B; CSC inversion ρ = −0.62, *p* < 0.05), and the positive alignment between codon stability coefficients and protein output only in the age-altered set (Figure 4D) are most consistent with weakened coupling between elongation dynamics and decay in aged oocytes. A larger fraction of messages gain stability with age, indicating that clearance of a GC-rich, actively translated subset slows with reproductive aging, extending their persistence during GV to MII (Figure 1B,C and Figure 3B). This altered decay rate could influence the precise timing of translation during oocyte maturation, particularly for transcripts involved in meiotic progression, and may contribute to the broader changes in mRNA abundance control observed in older oocytes [45]. The observation that aged oocytes retain certain transcripts for longer periods is consistent with the idea that post-transcriptional regulation is less tightly coordinated, which could have downstream consequences for protein production and cellular function. While these data do not identify the exact molecular cause, they provide a framework for investigating how age-related changes in RNA stability intersect with translational control to influence oocyte competence [46]. Our working hypothesis was that reproductive aging remodels translation-coupled mRNA decay via shifts in codon optimality. The present data support this: we observe age-dependent inversion of GC content-half-life relationships and a redefinition of codon optimality that together indicate altered coupling between translation and mRNA decay in aged oocytes.

Why do more transcripts gain stability with age? If translation-coupled decay is partially uncoupled, a GC-rich subset that is normally cleared can evade degradation and persist—consistent with the positive GC–protein association observed specifically in age-stabilized transcripts (Figure 3B). The likely consequences are temporal: extended translation windows may shift dosage and timing of proteins needed for meiotic progression and ZGA, with potential negative impacts on competence. Although initiation context may contribute, the codon-centric patterns here are most consistent with elongation-sensitive control (COMD). Finally, age-related changes in the inosine modification landscape (e.g., ADAR activity/distribution) could blunt GC-like marking for decay; this remains a hypothesis for future testing.

The shift from a negative to a positive correlation between GC content and half-life in transcripts altered by aging suggests a reorganization of mRNA decay control mechanisms. In transcripts unaffected by aging, the positive correlation between AU content and half-life suggests a model where AU-rich maternal mRNA are selectively stabilized for storage, consistent with patterns of translational dormancy [14,51,52,53]. Conversely, the negative correlation of GC content with stability in this group points to active, translation-coupled decay. A plausible, testable mechanism involves adenosine-to-inosine editing at wobble positions: because inosine is read as guanosine, inosine-enriched codons are functionally GC-like, a mark associated with selective clearance in oocytes [40]. Notably, inosine-modified transcripts can be cleared even when CNOT6L-mediated decay is impaired, consistent with a parallel, translation-coupled pathway. We hypothesize that reproductive aging alters ADAR activity/distribution or weakens coupling of inosine marks to CCR4–NOT/BTG4-driven decay, reducing targeting of GC-like/inosine-rich transcripts and contributing to the observed stability skew. Recent studies in human oocytes reveal that BTG4/CCR4-NOT activity impairment leads to accumulation of mRNA, potentially strengthening this hypothesis [8,41].

Relation to cross-species reproductively young baselines. Cross-species modeling [19] in young oocytes found GC content negatively associated with stability across mammals, whereas our study reveals that within reproductively aged human oocytes, these sequence–stability relations are reprogrammed, with GC features becoming positively associated with stability among age-altered transcripts. Thus, our results do not recapitulate species differences; they identify age-dependent remodeling of codon-linked decay within the human system. Within human oocytes, the direction of the GC–stability association inverts with age specifically in the age-altered set (Figure 2B,C), underscoring that our signal reflects reproductive aging, not species differences.

Among stabilized transcripts we observe enrichments in RNA processing, mitotic regulation, microtubule organization, and DNA repair, whereas destabilized transcripts are enriched for mitochondrial and tRNA-processing pathways (Figures S1 and S2). Conversely, the reduced stability of mitochondrial and tRNA-processing transcripts may impair energy production and translational capacity, both critical for maturation. These changes, while not definitive evidence of functional impairment, point to a redistribution of stability control that may influence oocyte developmental competence. The correlation inversion underscores a potential age-associated defect in the balance between storage and translation-linked decay [51]. Stabilization of GC-rich, translationally active mRNA is expected to widen the window of protein production during GV-MII, shifting dosage and timing for pathways we observe among the stabilized set (RNA processing, mitotic control, DNA repair). Such temporal mis-coordination could influence maturation dynamics and perturb the onset/robustness of ZGA, providing a plausible route by which reproductive aging reduces oocyte competence. These shifts target processes that set the timing and dosage of protein production during maturation, linking age-dependent stability to competence.

Across single-protein-group genes (*n* = 440), mRNA half-life and protein abundance are inversely related (ρ = −0.23, *p* < 0.05; Figure 3A), consistent with translation-coupled decay. These observations are consistent with translation-coupled decay mechanisms observed in oocytes and other systems [51]. In aged oocytes, GC-rich features are positively associated with protein abundance specifically among transcripts that gain stability (Figure 3B), indicating a shift toward persistence of translationally active messages. If GC-rich transcripts are both stable and associated with high protein levels, they may persist in a translationally active state for longer than intended. This extended activity could alter the temporal control of protein production during the GV-to-MII transition, potentially affecting processes that require tightly regulated bursts of synthesis. Conversely, the diminished GC–protein abundance association in transcripts with reduced stability suggests selective changes in how sequence features influence translation and decay. Together, these shifts point toward age-related rebalancing of mRNA fate decisions, where GC content plays an increasingly central role in determining both stability and translational output [51].

The contrasting codon composition of optimal sequences in unaffected versus aging-affected transcripts points to a fundamental shift in how codon usage influences stability with age. In unaffected transcripts, 19/20 optimal codons are AU rich (Figure 4A), whereas in age-altered transcripts 24/29 optimal codons are GC rich (Figure 4B), and the CSC profiles invert (ρ = −0.62, *p* < 0.05; Figure 4C). In young oocytes, the predominance of AU-rich optimal codons aligns with patterns observed in mammalian oocytes and cell lines, where AU-rich sequences are linked to storage and delayed translation [14,51,52,53]. This suggests that in the absence of aging, codon optimality supports a balance between stability for stored transcripts and targeted decay for actively translated ones. The codon-centric signals and the CSC–PACC relationships we report are most consistent with elongation-sensitive control (COMD), wherein slower elongation preferentially engages decay. That said, initiation context can also contribute. We therefore note that Kozak-sequence scores were included among recorded features in our pipeline (Methods, Section 4.2), and future work should directly test whether reproductive aging alters initiation control alongside elongation.

In aged oocytes, the enrichment of GC-rich optimal codons among stability-altered transcripts suggests that GC-rich sequences increasingly support stability rather than decay. This change may reflect impaired coupling between rapid translation and mRNA degradation, allowing GC-rich, actively translated transcripts to persist longer. The shift in the CSC–PACC relationship reinforces this interpretation: in young oocytes, higher translation rates, as measured by the correlation between codon usage and protein abundance differences (MII-GV iBAQ values; PACC), tend to coincide with lower stability (CSC), whereas in aged oocytes, higher translation rates and stability appear linked. This pattern is consistent with over-translation, extended translation of transcripts that would normally be cleared, a phenomenon reported in models of defective translational mRNA decay [14] and aged oocytes [6]. While the functional consequences remain to be fully characterized, these findings suggest that aging alters codon optimality rules in a way that could affect the timing and amplitude of protein synthesis during maturation. Together with the CSC–PACC sign flip (unaffected negative, affected positive; Figure 4D), these data indicate that reproductive aging rewires codon-linked stability rules within human oocytes.

These findings point toward a rebalancing of mRNA stability and translation control during reproductive aging. While the exact molecular drivers remain to be determined, the consistent involvement of GC content and codon composition suggests that age alters fundamental aspects of mRNA fate determination. Future studies using in vivo matured oocytes from healthy individuals across the reproductive lifespan will be important for clarifying whether these molecular patterns generalize beyond in vitro matured samples. In addition, future work should (i) quantify ribosome occupancy across age groups to test for persistent translation of stabilized GC-rich mRNA; (ii) probe deadenylation/CCR4-NOT activity and BTG4-axis integrity in aged oocytes; (iii) investigate whether the activity or abundance of ADAR enzymes, which mediate inosine modifications, is altered with reproductive aging; (iv) deploy codon-engineered reporter assays to causally link codon identity to decay in human oocytes; and (v) evaluate whether these molecular signatures predict clinical parameters in ART. In sum, reproductive aging appears to recalibrate codon-optimality rules, stabilizing a GC-rich subset of maternal mRNA and uncoupling translation from decay, a shift that likely perturbs maturation timing and early developmental competence. Collectively, these age-dependent shifts provide testable predictions for restoring proper clearance windows (e.g., by modulating elongation dynamics or inosine marking) and focus future work on mechanisms that recouple translation to decay in aged human oocytes. Practically, these results predict that recoupling translation to decay; for example, by restoring elongation-sensitive decay engagement, should compress the translation window of GC-rich maternal mRNA in aged oocytes.

Limitations. This work analyzes a single publicly available human IVM oocyte dataset (five donors) and draws correlative inferences without new experimental validation. Given the rarity of human oocytes, a focused human analysis is still informative, but findings should be interpreted within this scope and tested in future studies with in vivo–matured samples and direct measurements of ribosome occupancy, deadenylation, and inosine dynamics.

## 4. Materials and Methods

### 4.1. Alignment and Half-Life Calculation

Reproductively young and aged human GV and MII oocyte RNA-seq data were downloaded from the Short Read Archive project PRJNA377237 [45]. These poly(A) sequencing libraries were generated using SMARTer Ultra Low Input RNA HV kit (Clontech, Mountain View, CA, USA) using the manufacturer’s recommended protocol. Sequences were trimmed of known sequencing adaptors and quality scores using BBMap bbduk with the following settings; maq = 30 ktrim = l ktrim = r k = 23 mink = 11 hdist = 1 [54]. Read duplicates with upto 5 mismatches were removed using BBMap clumpify with the following settings; depupe sub = 5. Processed reads were aligned to Genecode v34 protein coding transcripts using kallisto v0.45 [55]. Kallisto data was processed in R for normalization and half-life determination [56]. Alignment statistics are provided in Supplemental File 1.

Transcript per million (TPM) counts were imported into R. Transcripts were eliminated if they had less than 1 TPM and were not reported in every sample for each age group (young GV *n* = 5, young MII *n* = 5, aged GV *n* = 5, aged MII *n* = 5). Filtered data was mean normalized and decay factor corrected as previously described [57]. Standard RNA-seq library normalization strategies such as DESeq and TMM, are inappropriate for RNA decay conditions as both approaches assume between 40 and 70% of genes are either increasing or decreasing in abundance and the total mass of RNA is relatively constant between conditions [58]. In mammalian oocytes, these conditions are not met as GV oocytes are transcriptional quiescent and more than 75% of mRNA is degraded by MII stage [14]. Decay factor normalization is necessary to account for the decrease in RNA-seq library complexity that occurs in the absence of transcription and the presence of active RNA decay. As a result, stable transcripts TPM values will increase in MII oocytes relative to GV oocytes [57]. To perform decay factor normalization, transcript abundance values were normalized to the mean of GV oocytes (time zero) for both reproductively young and aged samples, respectively. Next, decay factors were calculated by identifying mRNA whose mean normalized TPM fold increase was greater than one and whose variance across sample groups was less than the mean fold change. The decay factor fold change threshold was empirically determined using an iterative process that incrementally increased the fold change value by 0.5 until the variance of the decay factory normalization reached an inflection point, the fold change value of the inflection point was used as the threshold value for decay factory normalization. Mean normalized and decay factor corrected data were then fit to a linearized version of the following exponential decay model:

$$C\left(t\right)={C_{0}e}^{-\lambda t}$$

where $C\left(t\right)\;$ is the abundance of RNA at time, t . $C_{o}$ is the initial abundance of RNA at time zero. λ is the decay rate and t is time. Decay rates were used to calculate half-life values using the following formula:

$$t_{\frac{1}{2}}=\frac{l\left(0.5\right)\;}{\lambda }$$

The decay rate, λ *,* was calculated with the following formula:

$$\lambda =\frac{\sum_{i=i}^{n}\left(x_{i}-\bar{x}\right)\left(y_{i}-\bar{y}\right)}{{\left(x_{i}-\bar{x}\right)}^{2}}$$

where $x_{i}$ is the time value of the ${\;i}^{th}$ sample, $\bar{x}$ is the mean of all time, $y_{i}$ is the abundance value of the $i^{th}$ sample, and $\bar{y}$ is the mean of sample abundance. Standard error for the decay rate estimation was calculated with the following equation:

$${SE}_{\lambda }=\frac{\sigma^{2}}{\sqrt{\sum {\left(x_{i}-\bar{x}\right)}^{2}}}$$

where ${SE}_{\lambda \;}$ is the standard error of the estimated decay rate, $x_{i}$ is the time value of the ${\;i}^{th}$ sample, $\bar{x}$ is the mean of all time, and 2 is the standard deviation of the variance. The standard deviation of the variance was calculated using the below formula:

$$\sigma^{2}=\frac{\sum_{n=1}^{n}{\left(y_{i}-\bar{y}\right)}^{2}}{n-2}$$

where $y_{i}$ is the abundance value of the $i^{th}\;$ sample, $\bar{y}$ is the mean of sample abundance and n is the degree of freedom. P value estimates for the decay rate were generated by first calculating the t-statistic with the following formula:

$$t_{\lambda }=\frac{\lambda }{{SE}_{\lambda }}$$

To identify significantly different decay rates between young and aged samples, a standard score was calculated with the following formula:

$$Z=\frac{\lambda_{Y}-\lambda_{A}}{{SE}_{\lambda Y}+{SE}_{\lambda A}}$$

where Z is the standard score, $\lambda_{Yi\;}$ is the decay rate for a young transcript, $\lambda_{A\;}$ is the decay rate for an aged transcript, and ${SE}_{\lambda Y}$ and ${SE}_{\lambda A}\;$ are the standard error of variance for young and aged mRNA, respectively. The standard score was used to calculate Bonferroni corrected *p* values.

### 4.2. mRNA Feature, CSC, and Pathway Analysis

The Genecode v34 protein coding transcript FASTA reference was parsed in R to identify the sequence composition and lengths of CDS and UTR for each transcript. The universal motif Bioconductor package was used to identify GC (nnS, nSS, SSn, SSS)- and AU (nnW, nWW, WWn, WWW)-containing codons within the CDS of each transcript. The kozak motif (RYMRMVATGGC) was scored in Universalmotif [59,60]. The frequency of codons was calculated for each transcript using the Bioconductor biostrings package. Spearman and Pearson correlations between half-life values and mRNA features were performed in R. The codon correlation coefficient (CSC) was calculated as previously described [25]. The mRNA feature correlations and CSC for reproductive aging samples were calculated by binning the reproductively young half-life values into 1 h windows. The age half-life values were retrieved for each window, excluding the transcripts that did not increase or decrease more than 1 h in half-life values. Pathway analysis was performed using ReactomePA in R [61].

### 4.3. Mass Spectrometry Analysis

Mass spectrometry data for human GV and MII oocytes [49] were downloaded from ProteomeXchange Consortium (PXD003691). GV oocyte and MII samples were processed with MaxQuant (v1.6.17.0) and Andromeda search engine as previously described [49,62,63]. Briefly, the MS/MS spectra were used to search the Human UniProt database. Precursor mass tolerance for first pass and second pass were 20 ppm and 6 ppm, respectively, while the fragment mass tolerance was set to 0.5 Da and minimum peptide length set to 6. Quantification utilized unmodified unique and razor peptides. Modifications were set to fixed for cysteine carbamidomethylation and methionine oxidation. Trypsin/P was used for enzyme specificity with 2 miss cleavage events. A false discovery rate of 0.01 was used for both peptide and protein identifications. The protein identification was reported as a “protein group” if no unique peptide sequence to a single database entry was identified. Protein abundance was reported as iBAQ values [64]. In order to uniquely assign peptides to unique transcripts we focused on single protein producing genes. Accordingly, RNA–protein analyses in this study were limited to single-protein-group genes (*n* = 440) to ensure unambiguous transcript-to-protein mapping.

## Data Availability

All datasets analyzed in this study were previously published and are publicly available. The human oocyte RNA-seq data are deposited in the NCBI Sequence Read Archive (SRA) under BioProject PRJNA377237 [45], and the proteomics data used for GV-to-MII protein quantification are available via the ProteomeXchange Consortium under accession PXD003691 [49]. Reference resources (GENCODE v34 transcript annotations and the human UniProt proteome) are described in Methods.

## Supplementary Materials

The following supporting information can be downloaded at: https://www.mdpi.com/article/10.3390/ijms26199395/s1.

## Abbreviations

The following abbreviations are used in this manuscript:

ART	Assisted Reproductive Technology
CDS	Coding Sequence
COMD	Codon-Optimality-Mediated mRNA Decay
CSC	Codon Stability Coefficient
FACS	Fluorescence-Activated Cell Sorting
FDR	False Discovery Rate
GC	Guanine-Cytosine
GV	Germinal Vesicle
hCG	Human Chorionic Gonadotropin
HR	Hour
iBAQ	intensity-Based Absolute Quantification
IVF	In Vitro Fertilization
IVM	In Vitro Maturation
KS test	Kolmogorov-Smirnov test
MII	Metaphase II
MS	Mass Spectrometry
PACC	Protein Abundance Codon Correlation
Poly(A)	Polyadenylated
ppm	Parts Per Million
RNA	Ribonucleic Acid
RNA-seq	RNA Sequencing
TPM	Transcripts Per Million
UTR	Untranslated Region
ZGA	Zygotic Genome Activation
