# Correction: Brachova et al. Codon Composition in Human Oocytes Reveals Age-Associated Defects in mRNA Decay. *Int. J. Mol. Sci.* 2025, *26*, 9395

**DOI:** 10.3390/ijms262311694

**Published:** 2025-12-03

**Authors:** Pavla Brachova, Lane K. Christenson, Nehemiah S. Alvarez

**Affiliations:** 1 Department of Biomedical and Translational Sciences, Division of Woman and Child Health, Eastern Virginia Medical School, Macon and Joan Brock Virginia Health Sciences, Old Dominion University, Norfolk, VA 23518, USA; 2 Department of Cell Biology and Physiology, University of Kansas Medical Center, Kansas City, KS 66160, USA; 3 Advanced Sequencing Program, Eastern Virginia Medical School, Macon and Joan Brock Virginia Health Sciences, Old Dominion University, Norfolk, VA 23518, USA

## Missing Citation

In the original publication [1], *Tremblay, B.J.-M. Universalmotif: An R Package for Biological Motif Analysis. J. Open Source Softw. 2024, 9, 7012* was added because the bioinformatic tool Unversalmotif was used. The citation has now been inserted in Section 4. Materials and Methods, 4.2. mRNA Feature, CSC, and Pathway Analysis, paragraph 1, and should read as follows:

The kozak motif (RYMRMVATGGC) was scored in Universalmotif [59,60].

With this correction, the order of some references has been adjusted accordingly. 

## Text Correction

There was an error in the original publication. The citation numbers of the references in the Data Availability Statement section are incorrect.

A correction has been made to “All datasets analyzed in this study were previously published and are publicly available. The human oocyte RNA-seq data are deposited in the NCBI Sequence Read Archive (SRA) under BioProject PRJNA377237 [45], and the proteomics data used for GV-to-MII protein quantification are available via the ProteomeXchange Consortium under accession PXD003691 [49]. Reference resources (GENCODE v34 transcript annotations and the human UniProt proteome) are described in Methods”.

The authors state that the scientific conclusions are unaffected. This correction was approved by the Academic Editor. The original publication has also been updated.
