# Supplementary figures and images for: Codon Composition in Human Oocytes Reveals Age-Associated Defects in mRNA Decay
Source: Int J Mol Sci. 2025 Sep 26;26(19):9395. doi: 10.3390/ijms26199395 (PMC12524513; doi:10.3390/ijms26199395)

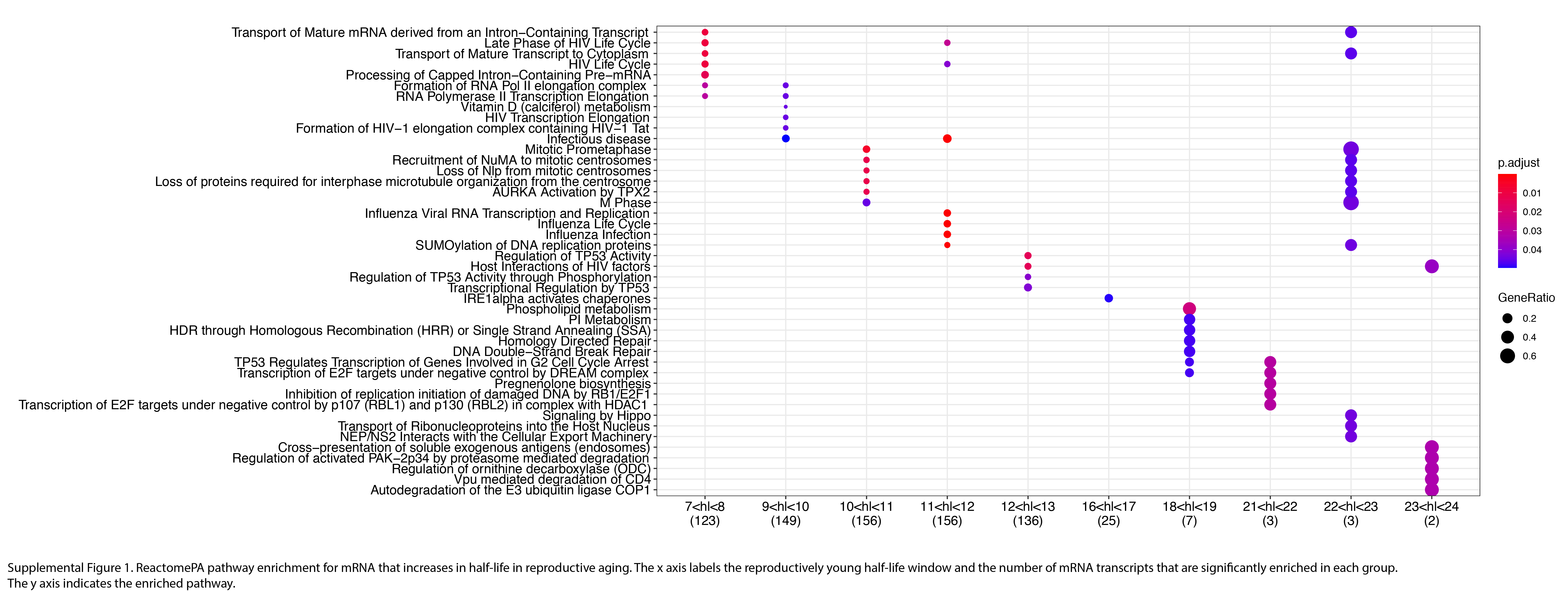

Supplement: Supplementary file 1 [file ijms-26-09395-s001.zip › Supplemental Figure_S1.jpg]

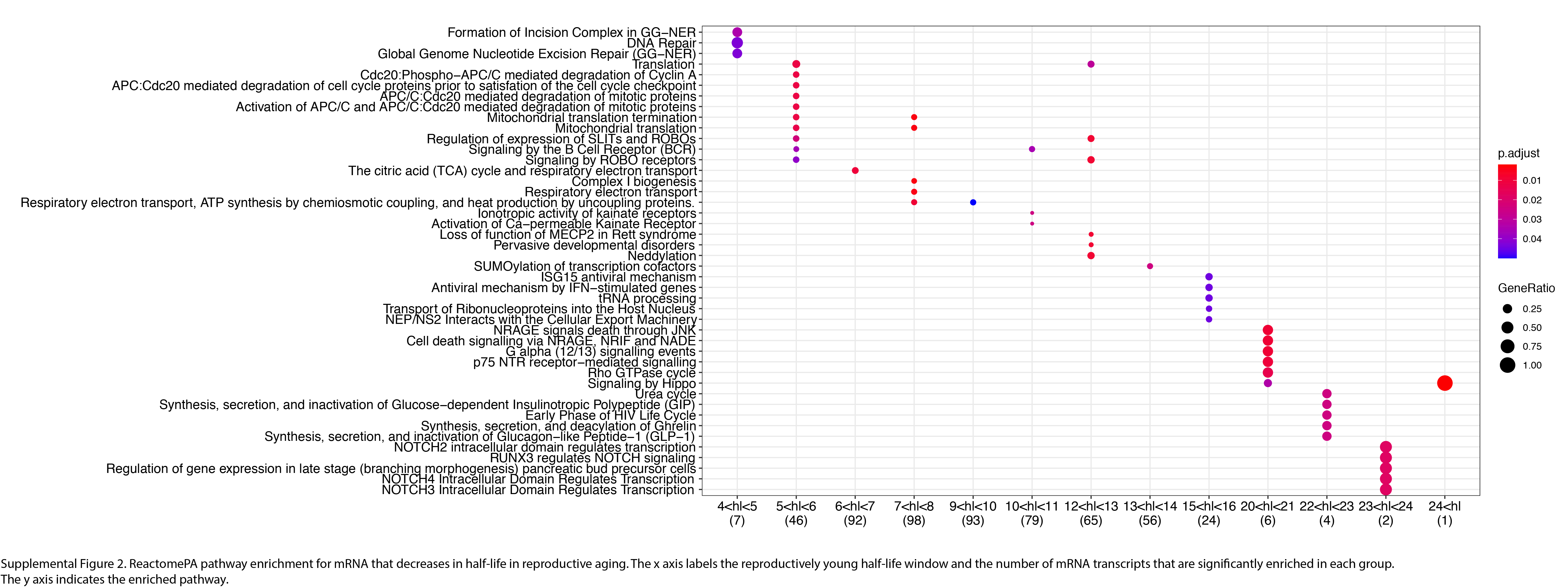

Supplement: Supplementary file 1 [file ijms-26-09395-s001.zip › Supplemental_Figure_S2.jpg]
